# Barcelona Clinic Liver Cancer Stage B Hepatocellular Carcinoma: Transarterial Chemoembolization or Hepatic Resection?

**DOI:** 10.1097/MD.0000000000000180

**Published:** 2014-12-05

**Authors:** Lei Jianyong, Yan Lunan, Wang Wentao, Zeng Yong, Li Bo, Wen Tianfu, Xu Minqing, Yang Jiaying

**Affiliations:** From the Department of Liver Surgery (LJ, YL, WW, ZY, WT, XM); General Surgery (LB); and Transplantation Center, West China Hospital of Sichuan University, Chengdu, China (YJ).

## Abstract

According to the Barcelona Clinic Liver Cancer (BCLC) guidelines, transarterial chemoembolization (TACE) is recommended for BCLC stage B hepatocellular carcinoma (HCC). However, an investigation of the use of resection for BCLC stage B is needed. Therefore, we compared the efficacy and safety of hepatic resection (HR) with that of TACE in treating intermediate HCC.

We retrospectively enrolled 923 patients with BCLC stage B HCC who underwent TACE (490 cases) or HR (433 cases). The baseline characteristics, postoperative recoveries, and long-term overall survival rates of the patients in these 2 groups were compared. Subgroup analyses and comparisons were also performed between the 2 groups.

The baseline demographic and tumor characteristics, in-hospital mortality rate, and 30-day mortality rate were comparable between the 2 groups. However, the patients in the resection group suffered from more serious complications compared with those in the TACE group (11.1% vs 4.7%, respectively, *P* < 0.01) as well as longer hospital stays (*P* < 0.05). The resection patients had significantly better overall survival rates than the TACE patients (*P* < 0.01). In the TACE group, patients with Lipiodol retention showed much higher 1-, 3-, and 5-year overall survival rates than those in the noncompact Lipiodol retention group (*P* < 0.01). Subgroup analyses revealed that patients with 1 to 3 tumor targets showed much better 1-, 3-, and 5-year overall survival rates in the resection group (*P* < 0.01), but no difference was observed for the patients with >3 targets.

Our clinical analysis suggests that patients with BCLC stage B HCC should be recommended for resection when 1 to 3 targets are present, whereas TACE should be recommended when >3 targets are present.

## INTRODUCTION

Hepatocellular carcinoma (HCC) accounts for 80% to 90% of primary liver cancers; it is the fifth most common cancer worldwide for men and the seventh for women.^1^ The prognosis for HCC patients is determined by tumor status, liver function reserve, general health, and treatment efficacy.^2^ More than 80% of HCC patients present with cirrhosis; only 10% to 15% of these patients have potentially resectable tumors.^3^ Liver transplantation (LT), surgical resection, and radiofrequency ablation (RFA) are the 3 potential radical treatments for early-stage HCC patients.^4^ LT results in the widest possible resection margin, removes the underlying cirrhotic liver, and restores hepatic function; thus, it should be considered as the most effective treatment for these patients. However, the liver donor pool is small. Thus, hepatic resection (HR) represents a popular alternative treatment for early-stage HCC. The treatment outcomes for HCC patients are affected by multiple variables, including tumor burden, the Child-Pugh score of liver function reserve, and the performance status of the patient.^5^ In 1996, the Barcelona Clinic Liver Cancer (BCLC) classification staging system was developed to account for these 3 variables by establishing BCLC groups.^6,7^ The BCLC staging system was recently utilized to stratify HCC patients according to the guidelines established by the American Association for the Study of Liver Disease.^8^ The stratification capacity of the BCLC system for predicting prognosis has been cross-validated in several cohorts of HCC patients.^9,10^ In addition to estimating prognosis, the main advantage of the BCLC staging system is that it establishes links between staging and treatment indications.^11^ This staging system recommends different treatment options for each stage of the disease,^7^ stating that transarterial chemoembolization (TACE) should be considered for patients with BCLC-B HCC and certain patients with BCLC-C HCC. Curative HR is indicated only for patients with early-stage HCC and satisfactory liver function (BCLC-A).^12^ Although the BCLC staging system classifies patients with a single nodule as BCLC-A, according to the Milan criteria, a solitary target with a diameter of >5 cm should be excluded from LT, and RFA should not be recommended when the diameter of a solitary target is >5 cm. Notably, not all solitary HCCs can be resected (such as those >10 or 15 cm). Thus, in our study, we defined the BCLC-B stage as patients with Child scores of A or B with large, single-focus HCC (>5 cm) and those with multifocal HCC, which is defined as >3 tumors of any size or 2 to 3 tumors with a maximal diameter of >3 cm in the absence of vascular invasion or extrahepatic spread.^13^

The indication of liver resection for BCLC-B HCC remains controversial, although it has been assessed in many studies.^14^ The guidelines from the European Association for the Study of Liver Disease and the American Association for the Study of Liver Disease do not recommend HR for treating BCLC stage B/C HCC.^2,7^ However, many studies conducted in North America, Europe, and Asia have proposed that liver resection can be safely performed in patients with large or multinodular HCC and good liver function,^15–17^ and some reports have advocated that BCLC stage C HCCs are also candidates for HR.^12^ Therefore, using a large cohort of patients from West China, we compared the efficacy and safety of TACE with that of HR for treating BCLC stage B HCC.

## METHODS

**FIGURE 1:**
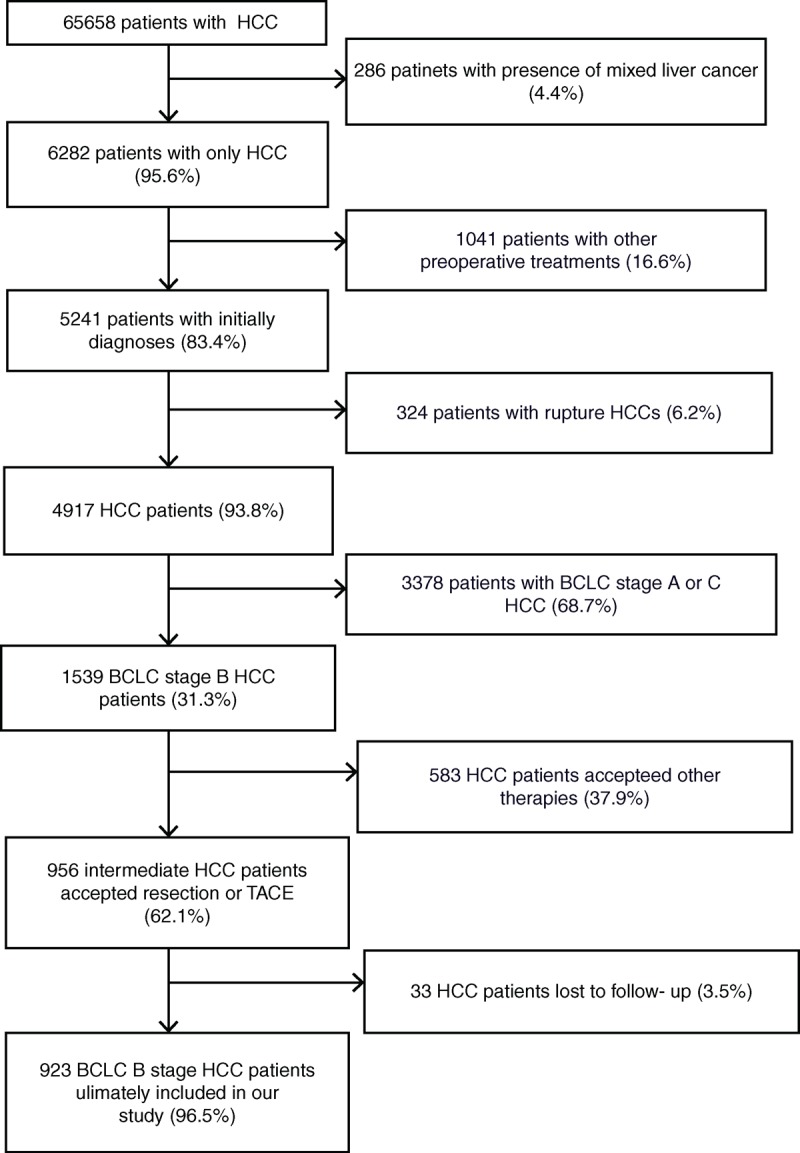
The databases included 6568 HCC patients. Based on the inclusion and exclusion criteria, 923 HCC patients were selected for baseline-adjusted analyses. HCC = hepatocellular carcinoma.

Between July 2002 and November 2008, 6568 patients were diagnosed with HCC and underwent treatment at the West China Hospital of Sichuan University. In the present study, we only included intermediate HCC patients with Child-Pugh A or B scores who underwent liver resection or TACE. Patients with other initial treatments for HCC, extrahepatic metastasis, or diminished liver function (Child C) were excluded from our study. Patients who underwent LT, RFA, or other therapies were also excluded. Based on the inclusion and exclusion criteria, 923 patients (14.1%) were enrolled in this retrospective study (Figure 1). We divided the 923 patients into 2 groups based on the therapy administered as follows: the liver resection group (n = 433) and the transarterial chemoembolization group (n = 490). Next, we retrospectively analyzed the 2 groups to compare long-term outcomes in terms of 1-, 3-, and 5-year overall survival rates. The ethical conduct of this study was approved by our departmental review board (West China Hospital of Sichuan University) in agreement with the 1990 Declaration of Helsinki and subsequent amendments, and all patients signed informed consent before TACE or resection.

HCC diagnosis in the TACE group was based on 2 imaging scans (contrast-enhanced ultrasound, double-phase helical computed tomography scan, or magnetic resonance imaging) and serum alpha-fetoprotein (AFP) levels. For the resection patients, HCC was diagnosed by retrospective histopathological examination. BCLC stage B was defined as the presence of 1 lesion of >5 cm in diameter or of 2 to 3 lesions (of which at least 1 was >3 cm in diameter) or of >3 lesions of any diameter. Portal hypertension was defined as the presence of esophageal variance or a platelet count of <100 × 10^9^ cells/L in association with splenomegaly.^12^ HCC resection was performed anatomically by an experienced surgeon, leaving at least 1 cm of nontumor margin. All surgical procedures were performed with the patient under general anesthesia and were guided by ultrasound. Anatomical resection was based on the segmental division of the liver, but nonanatomical resection using a sufficient resection margin was often adopted to ensure that an adequate volume of the liver remained.^18^ The TACE protocol included the superselective technique, in which tumor-feeding arteries were catheterized with a highly flexible coaxial microcatheter passing through a 4-Fr catheter that had been previously placed within the hepatic artery. A mixture of epirubicin and Lipiodol (average total volume of 50 mL) was then injected under fluoroscopic control, followed by embolization with Spongel particles until vessel stasis was achieved.^4,19^ The efficacy of liver resection or TACE was evaluated using enhanced imaging scans after 1 and 2 to 3 postoperative months, and AFP levels were measured every 2 to 3 months. The therapy used to treat HCC recurrence, such as re-resection, RFA, re-TACE, or LT was dependent upon the patient's wishes in addition to their liver function, tumor characteristics, and the availability of a liver graft for LT.

Categorical variables were compared using the *χ*^2^ and 2-tailed Fisher exact tests. Continuous variables were compared using the Mann–Whitney *U* test. The effects of resection and TACE were compared by univariate analysis. Multivariate analysis was performed using Cox regression hazard analysis to calculate the hazard ratios and *P* values of independent variables for overall survival. The continuous data were expressed as the median values with interquartile ranges. Survival curves were estimated using the Kaplan–Meier method and compared using the log-rank test. A 2-tailed *P* < 0.05 was considered to be statistically significant. All statistical analyses were performed using the SPSS 17.0 statistical package (SPSS Inc, Chicago, IL).

## RESULTS

### Baseline Characteristics of Patients and Tumors

Among the 923 patients with BCLC stage B HCC enrolled in this study, 433 received liver resection and 490 received TACE. Procedures were typically performed by the attending physician based on the tumor characteristics, liver function, and the patients’ condition and religious beliefs. Using the BCLC staging system, we defined BCLC stage B as the presence of one lesion of >5 cm in diameter, 2 to 3 lesions (of which at least 1 was >3 cm in diameter), or >3 lesions of any diameter. We defined portal hypertension as the presence of esophageal variance or a platelet count of <100 × 10^9^ cells/L in association with splenomegaly. Most of the patients in both groups showed portal hypertension because of the high prevalence of hepatitis B virus (HBV) or hepatitis C virus (HCV) infection. For all patients, the shortest follow-up period was 5 years. Table 1 shows the demographics and clinical characteristics of the patients in both groups. Patients who underwent liver resection had an older average age and greater body mass index, but these differences were not statistically significant. No significant between-group differences were observed in patient sex, weight, height, or cirrhosis etiology. Most patients in both groups showed good liver function (Child A), and no difference was observed in the model for end-stage liver disease score or the Child Score. The majority of patients in both groups exhibited a good performance status (grade 0), but 118 patients in the resection group and 122 patients in the TACE group presented with symptoms that were related to tumor burden (Eastern Cooperative Oncology Group [ECOG] PS 1-2).

**TABLE 1:**
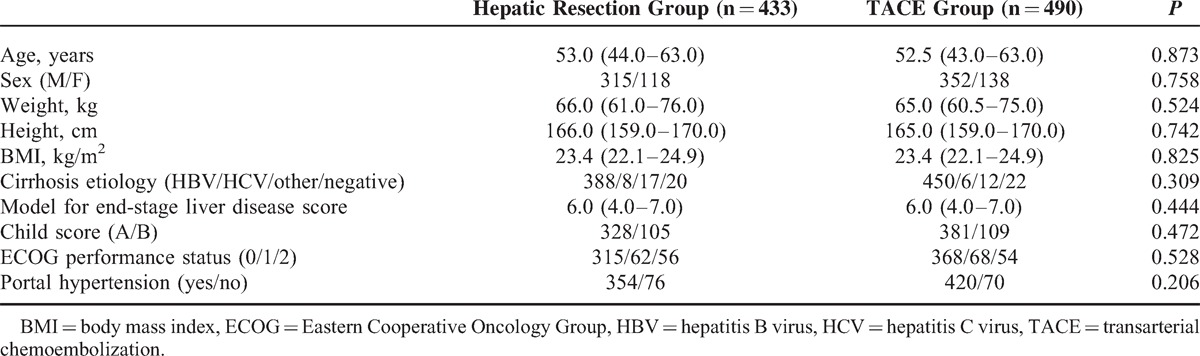
Baseline Characteristics of the Patients Submitted to Hepatic Resection or TACE

As illustrated in Table 2, the average tumor target number per patient in the TACE group was greater than that found in the resection group, and the diameter per target was larger in the TACE group; however, these differences were not statistically significant (*P* > 0.05). According to the BCLC staging system, we divided the 2 groups into 3 subgroups as follows: single target, 2 to 3 targets, and multiple targets. Single-target patients displayed a slightly smaller target diameter in the resection group compared with the TACE group (*P* = 0.060), and the differences between the TACE and 2- to 3-target (*P* = 0.065), and multiple target (*P* = 0.137) groups were also not statistically significant. Regarding the preoperative AFP level, which is a biomarker for HCC, we divided all patients into 4 groups according to AFP level and found no significant difference between the TACE and resection groups (*P* = 0.072).

**TABLE 2:**
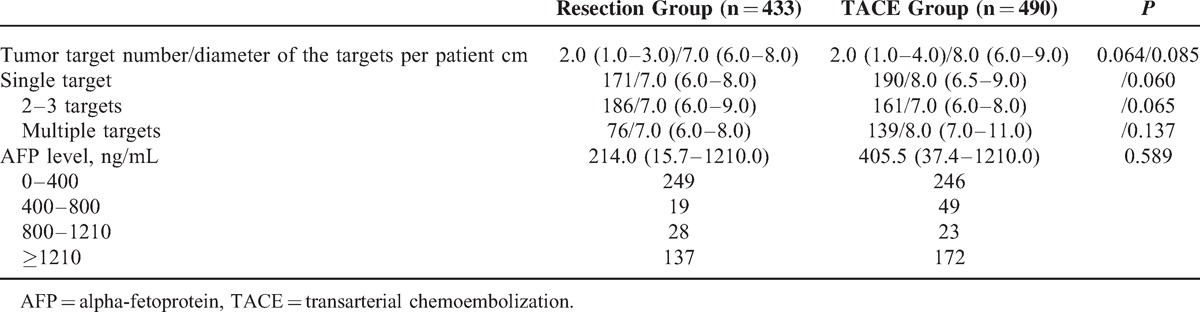
Comparison of Tumor Characteristics

### Mortality, Morbidity, and Postoperative Outcomes

The in-hospital mortality rate was 1.6% in the resection group and 1.0% in the TACE group (*P* = 0.425). No significant differences were observed between the 2 groups for 30-day mortality (2.3% and 2.0% in the resection and TACE groups, respectively) (*P* = 0.780). The Clavien–Dindo classification of surgical complications was used to assess postoperative complications. No significant difference was observed in the postoperative complication rate between the 2 groups (*P* = 0.771, Table 3). However, when we compared minor (grade I–II) and serious (grade III-V) complications, the resection group displayed a significantly greater number of serious complications (4.7% vs 11.1%, respectively, *P* < 0.01) and a lower number of minor complications (15.2% vs 23.9%, respectively, *P* = 0.001) compared with the TACE group. In a subgroup analysis, the complication rates of grade I, IIIa, IVa, IVb, and V were not significantly different, but the TACE group had a higher prevalence of grade II (13.7% vs 6.5%, respectively, *P* < 0.01) and a lower prevalence of grade IIIb (0.8% vs 2.5%, respectively, *P* = 0.039) complications compared with the resection group. The most common complication reported by the grade II patients was embolism syndrome (49 cases), which included fever, vomiting, and pain (Table 3).

**TABLE 3:**
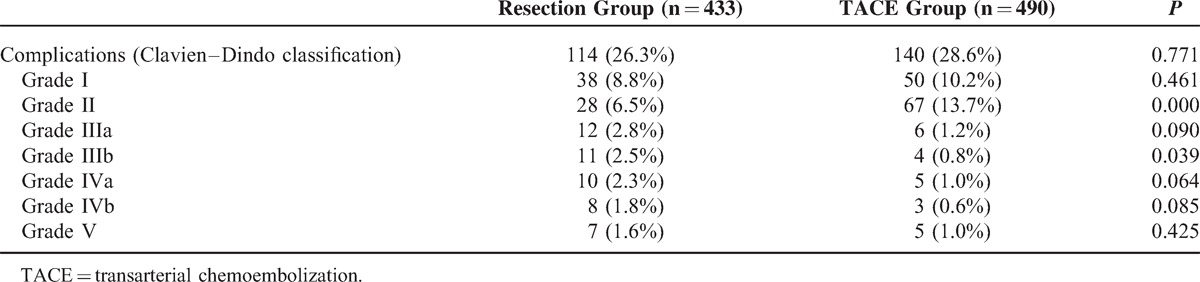
Postoperative Recovery of the 2 Patient Groups

The median hospital stay in the resection group was 9.0 (5.0–12.0) days, which was much longer than that of the TACE group (4.0, 3.0–5.0 days) (*P* < 0.01). The median number of days required for the patients in the 2 groups to return to a normal work schedule was not significantly different (23.0, 16.0–32.0 vs 18.0, 12.0–26.0, respectively, *P* = 0.143).

### Comparison of the Between-group Overall Survival Rates

**FIGURE 2:**
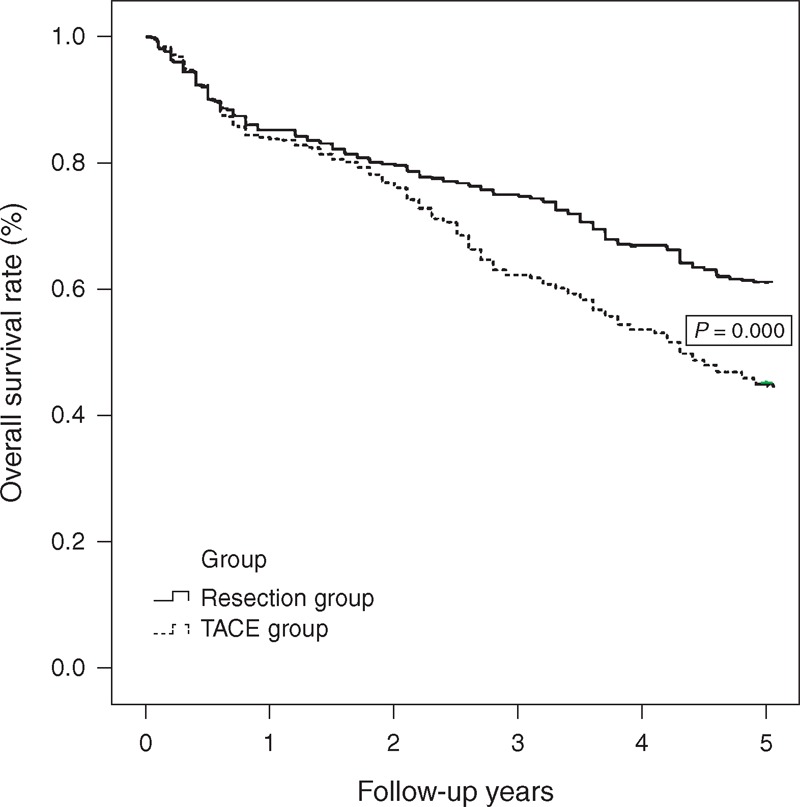
Overall survival curves for BCLC stage B HCC patients who received TACE or resection. Patients in the TACE group showed 1-year survival rates similar to those of the patients in the resection group (>0.05). However, a more marked benefit of resection on overall survival was observed at 3 and 5 years following treatment (P0.000). These between-group differences increased dramatically over time. BCLC = Barcelona Clinic Liver Cancer, HCC = hepatocellular carcinoma, TACE = transarterial chemoembolization.

As shown by at least 5 years of follow-up data, the patients in the resection group displayed significantly better overall survival rates than those in the TACE group (Figure 2, *P* < 0.01). However, the TACE and resection groups showed similar survival rates at 1 year posttreatment (84.1% vs 85.2%), whereas the benefit of resection on overall survival was more obvious at 3 years (71.1% vs 62.2%, respectively) and even more so after 5 years (61.2% vs 45.1%, respectively). As shown in the Figure 2, the differences between the 2 groups became more dramatic as time progressed. During the 5-year follow-up period, the patients in the resection group who died had a median overall survival time of 24.8 months, whereas those in the TACE group had a median overall survival time of 26.9 months (*P* = 0.231). The most common cause of death was tumor recurrence (85.1% in the resection group and 81.8% in the TACE group), followed by liver failure. The 1-, 3-, and 5-year tumor recurrence rates were 11.3%, 20.1%, and 32.3%, respectively, in the resection group.

**FIGURE 3:**
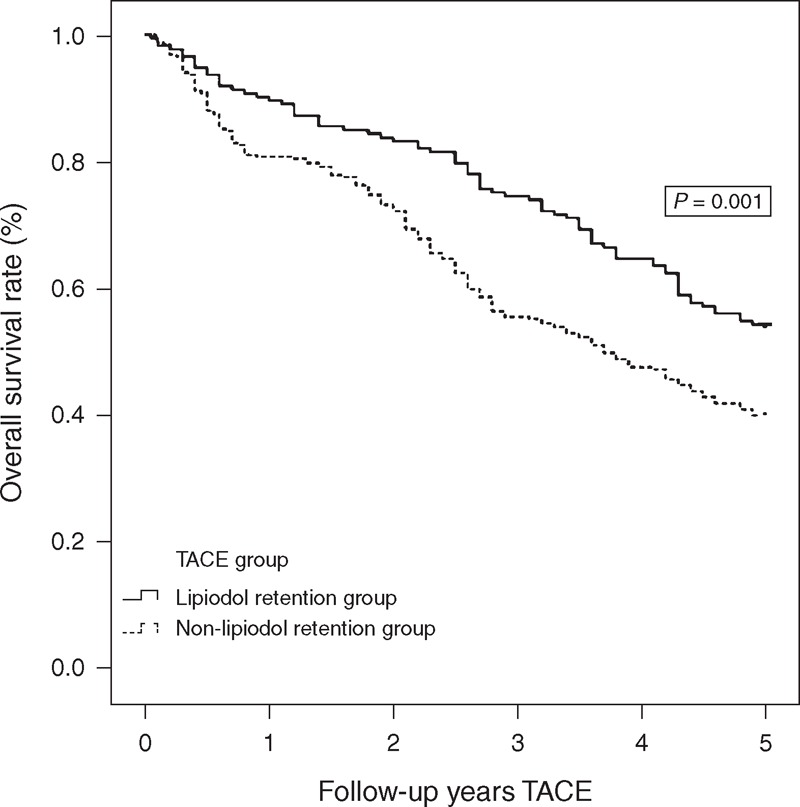
Overall survival curves of subgroups of BCLC stage B HCC patients who received TACE or resection. The overall survival rate of patients with Lipiodol retention was much greater than that of the noncompact Lipiodol retention group (*P* = 0.001). BCLC = Barcelona Clinic Liver Cancer, HCC = hepatocellular carcinoma, TACE = transarterial chemoembolization.

The 490 patients in the TACE group were divided into 2 groups according to their compact Lipiodol pattern, including the Lipiodol retention group (173 patients) and non-Lipiodol retention group (317 patients). Lipiodol labeling was considered to be compact when the oily contrast medium was clearly visible and well dispersed throughout the tumor, and it was considered to be noncompact in all other cases. When we compared the long-term outcomes of these 2 groups, the overall survival rate of the patients with Lipiodol retention was much greater at 1, 3, and 5 years after treatment compared with the noncompact Lipiodol retention group (Figure 3, *P* = 0.001).

### Subgroup Analysis by Tumor Number

**FIGURE 4:**
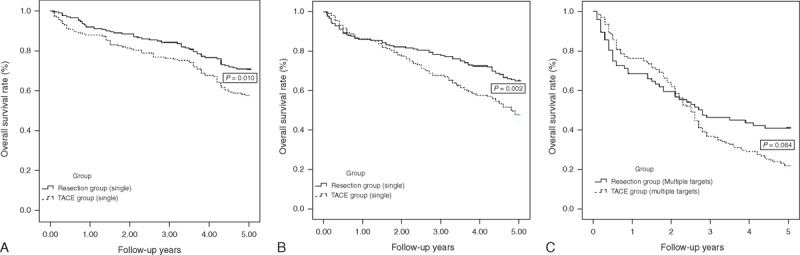
(A–C) Overall survival curves of subgroups of BCLC stage B HCC patients who received TACE or resection according to tumor target number: The benefit of resection on overall survival was much more obvious in the HCC patients with 1 to 3 targets (*P* < 0.05), but there was no significant difference in patients with >3 HCC targets (*P* = 0.064). BCLC = Barcelona Clinic Liver Cancer, HCC = hepatocellular carcinoma, TACE = transarterial chemoembolization.

Patients in the 2 groups were divided into 3 subgroups according to their BCLC stage B classifications as follows: 1 lesion of >5 cm in diameter, 2 to 3 lesions (of which at least 1 was >3 cm in diameter), or >3 lesions of any diameter. First, we compared the survival rates of the patients with only 1 tumor target (171 patients in the resection group and 190 patients in the TACE group). The 1-, 3-, and 5-year overall survival rates for the 171 patients in the resection group were 91.8%, 84.2%, and 70.8%, respectively, which were significantly greater than those of the 190 patients in the TACE group (87.9%, 76.3%, and 57.9%, respectively) (Figure 4A, *P* = 0.010). For the BCLC stage B HCC patients with 2 to 3 tumor targets, the 1-, 3-, and 5-year overall survival rates were 86.5%, 78.5%, and 65.1%, respectively, in the resection group and 86.3%, 67.7%, and 50.3%, respectively, in the TACE group (Fig. 4B, *P* = 0.002). However, the overall survival rates of the HCC patients with multiple tumors (>3 targets) did not significantly differ between the groups (1-, 3-, and 5-year overall survival rates of 68.4%, 46.0%, and 40.8%, respectively, in the resection group and 76.3%, 36.7%, and 21.6%, respectively, in the TACE group) (Fig. 4C, *P* = 0.064).

### Univariate and Multivariate Analyses

Univariate survival analysis was performed using 15 of the following variables (Table 4): age, sex, Child score, ECOG performance, portal hypertension, tumor number, largest tumor size, overall tumor size, AFP level, HBV infection, treatment method, platelet count ×10^9^ cells/L, WBC count ×10^9^ cells/L, and neutrophil-to-lymphocyte ratio (NLR). Portal hypertension, multiple tumor targets, overall tumor size of >10 cm, AFP level of >400 ng/mL, TACE treatment, and NLR of >2.81 were predictors of survival in the total population. Multivariate analysis demonstrated that portal hypertension (hazard ratio [HR] = 1.218, 95% confidence interval [CI] 1.002–1.343, *P* = 0.010), >3 tumor targets (HR = 1.116, 95% CI 1.024–1.231, *P* = 0.028), AFP level of >400 ng/mL (HR = 1.231, 95% CI 1.115–1.453, *P* = 0.023), TACE treatment (HR = 2.221, 95% CI 1.781–3.021, *P* < 0.01), and NLR of >2.81 (HR = 1.008, 95% CI 0.991–1.211, *P* = 0.031) were predictors of overall survival for the BCLC-B HCC patients.

**TABLE 4:**
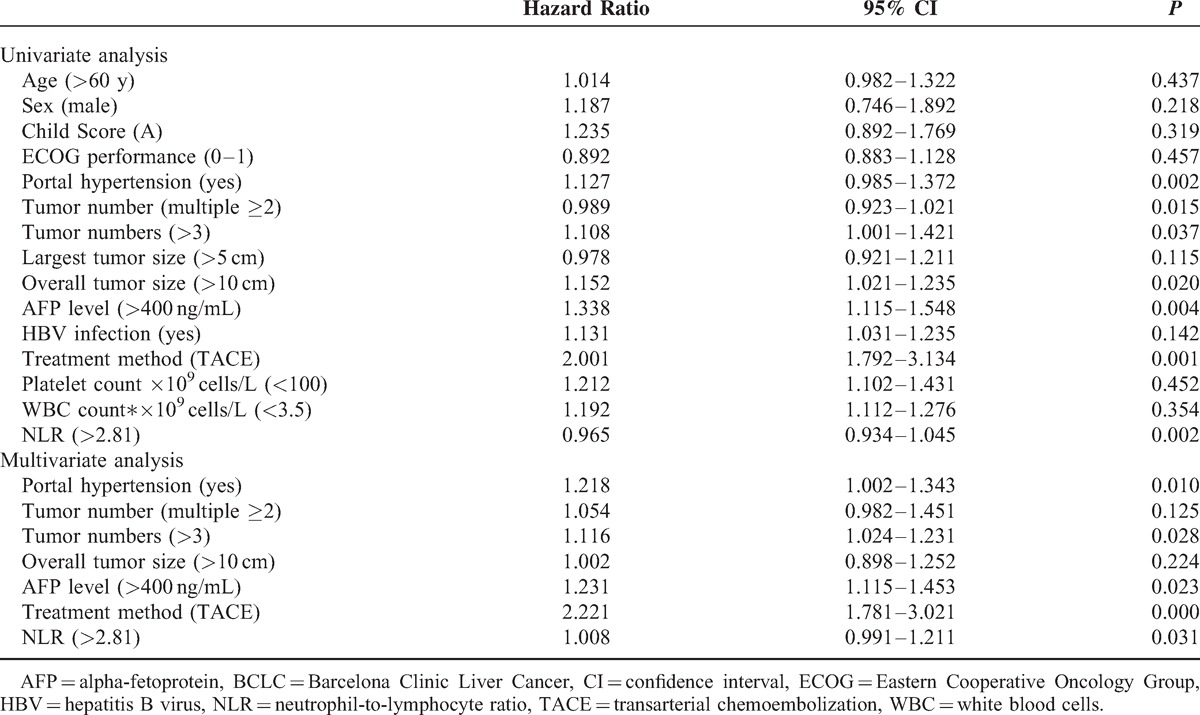
Univariate and Multivariate Analyses of Overall Survival in BCLC Stage B Patients

### Follow-up Treatment

**FIGURE 5:**
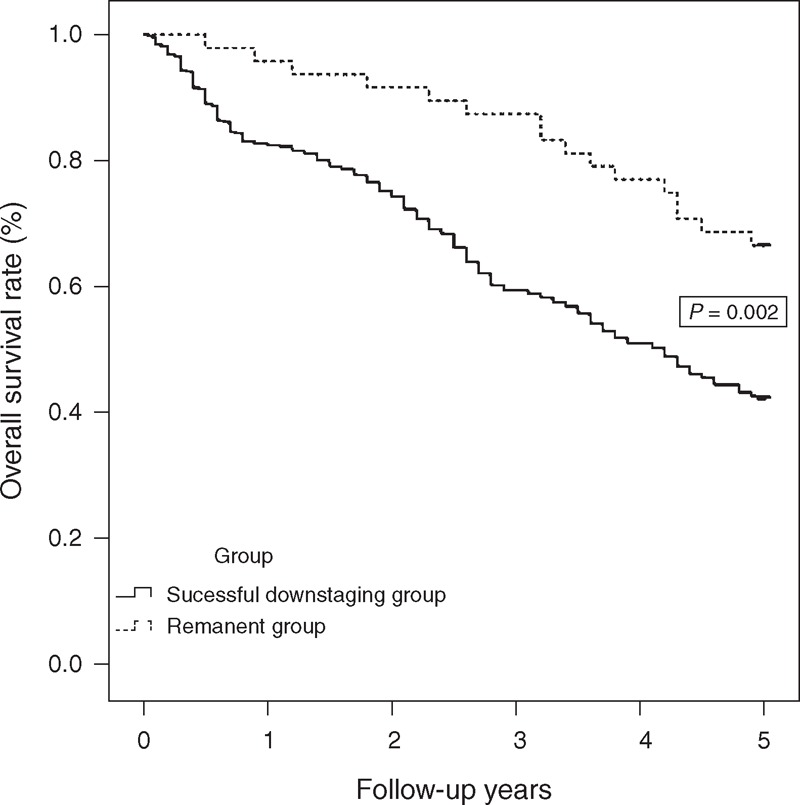
Overall survival curves of patients with successful downstaging who received TACE. The HCC patients who underwent successful TACE showed much better overall survival rates compared with the other patients (*P* = 0.002). HCC = hepatocellular carcinoma, TACE = transarterial chemoembolization.

The follow-up analyses indicated that among treatments administered after initial TACE treatment or resection, including re-TACE, re-resection, high-intensity focused ultrasound, LT, RFA, alcohol injection, transarterial chemoinfusion, gamma knife, and sorafenib, the most common follow-up treatment was TACE. No significant difference was observed between the 2 groups (*P* = 0.216). In the TACE group, the tumors of 48 (9.8%) patients were successfully downstaged to BCLC stage A, and radical therapies, including LT if liver graft was available, resection, or RFA, were considered to be acceptable. Regarding the overall survival rates between the groups, the 1-, 3-, and 5-year survival rates were significantly higher in the downstaged patients (95.8%, 87.5%, and 66.7%, respectively) compared with the remaining HCC patients (82.8%, 59.5%, and 42.8%, respectively) (Figure 5, *P* = 0.002).

## DISCUSSION

Numerous staging systems have been proposed for the prognostic classification of HCC, including the Okuda^20^ system, tumor node metastasis,^21^ Cancer of the Liver Italian Program,^22^ Japan Integrated Staging,^23^ Chinese University Prognostic Index,^24^ and BCLC staging system.^6^ Because the BCLC staging system links 5 different HCC stages with the appropriate therapeutic treatment options, it is endorsed by the European Association for the Study of the Liver^2^ and the American Association for the Study of Liver Diseases,^8^ and it has been widely adopted as the staging system of choice in many countries. The BCLC-proposed treatment for intermediate-stage HCC suggests TACE as the first-line therapy because it presents with the most improved 2-year survival rate compared with more conservative treatment methods. HR for BCLC stage B HCC is considered to be a poor option associated with an unfavorable prognosis.^25^ However, some studies have demonstrated good results after resection in BCLC B HCC patients. Moreover, according to AASLD guidelines, TACE is the first-line therapy recommended for intermediate-stage patients; however, in select cases, alternative treatment options and strategies, including surgery, should be considered.

In the present study, most HCC cases (91%) were related to HBV infection, which is similar to other reports on Chinese patients. A majority of patients in both groups (83.9%) showed portal hypertension due to HBV or HCV infection. Several reports have indicated that elderly patients generally prefer the less-invasive TACE procedure.^26^ The mean age in this study was comparable between the 2 groups, and most of the HCC patients in our study were younger than those in other reports. Few elderly HCC patients were included in our study because of the lack of medical security for the elderly in China compared with other developed countries.^27–30^

We also evaluated and compared postoperative complications using the Clavien–Dindo classification system. Although there was a comparable postoperative complication rate between the 2 groups, the resection group displayed a much greater rate of serious complications and fewer minor complications. The following reasons could have accounted for this imbalance: first, all resection procedures were performed with the patients under general anesthesia, whereas local anesthesia was used for the TACE patients; second, the resection procedure is much more invasive and risky compared with TACE;^31^ and third, some serious complications, such as postoperative bleeding and biloma, were specific to resection, whereas embolism syndromes were specific to TACE and were minor (grades I or II).^32^ These factors may have led to longer hospitalization stays for the patients in the resection group.

In the present study, 48 patients who underwent successful TACE as downstaging therapy followed by radical therapy showed a better overall survival rate. Chemoembolization involves the mixing of iodized oil with ≥1 anticancer drugs, such as doxorubicin hydrochloride, epirubicin hydrochloride, mitomycin C, cisplatin, neocarzinostatin, or floxuridine. The mixture is then injected into tumor-feeding vessels, and the vessels are embolized with gelatin sponges. In our series, the main anticancer drug used for chemoembolization was epirubicin. TACE remains as the most commonly used palliative therapy for unresectable HCC, and it has been evaluated as a potentially effective downstaging modality in Europe^33^ and the United States.^34^ Thus, it is the recommended treatment strategy for patients with advanced HCC, according to the American Association for the Study of Liver Diseases guidelines.^8^ Regarding the use of LT for HCC, patient response to TACE as a dynamic criterion is readily identifiable in clinical practice, and it appears to reflect tumor biological properties and aggressiveness.^35^ Response criteria, such as descriptions of the size and number of nodules, have been shown to be more reliable in predicting HCC recurrence compared with the Milan or University of California,San Francisco (UCSF) criteria.^36–38^ mRECIST (complete response, partial response, progressive disease, and stable disease) as assessed by imaging scans after TACE is a reproducible and reliable method for differentiating responders from nonresponders.^38^ Moreover, as described in the present report, tumors with Lipiodol retention are more susceptible to extensive tumor necrosis after TACE. Numerous additional studies have demonstrated similar results. Patients exhibiting compact uptake of Lipiodol into their tumor had a greater probability of survival than those with less compact uptake.^26^ Therefore, the Lipiodol uptake pattern after TACE can be considered to be a posttreatment prognostic marker that is comparable with mRECIST. Although several groups have argued that preoperative TACE complicates surgery because of chemical hepatitis of the hepatic parenchyma, edema, hemorrhage, and adhesions, a recent review has indicated that there are no increases in morbidity and mortality after TACE in patients who have undergone resection.^41^ Pre-TACE can also reduce surgical risk by shrinking macroscopic tumor size, enlarging the remaining liver, clarifying the tumor margin and tumor number, and controlling or eliminating micrometastases.^39^ Thus, tumor response to TACE may be a useful criterion for selecting optimal candidates for HR and for avoiding unnecessary invasive surgery.

Previous randomized controlled trials have demonstrated that TACE is superior to symptomatic treatment in terms of overall survival rate.^40^ TACE is used as a palliative therapy for unresectable HCC, and previous studies have shown that patients with unresectable HCC, poor liver function, and multiple liver tumors or relatively large tumor sizes are eligible for this procedure.^41^ Our results revealed that patients in the resection group demonstrated superior survival rates compared with those in the TACE group with 1 to 3 BCLC stage B tumor targets. This finding may have been due to the superiority of HR relative to TACE in tumor ablation, although HR is far more invasive.^42^ However, the survival rate did not significantly differ between the TACE group and the HR group for BCLC B stage HCC with multiple tumor targets (>3 targets). Most cases of HCC with multiple tumor targets are caused by the spread of intrahepatic metastasis and micrometastases from the primary tumor, and it is unsurprising that initial treatment with hepatectomy fails to cure the majority of these patients.^43^ The better outcome indicated by this study compared with other reports on the resection of BCLC-B HCC^26,43^ is mainly due to our strict inclusion criteria, and, in particular, the increased number of patients with a solitary tumor target with a diameter of >5 cm. Downstaging with TACE and additional treatment options can allow for subsequent resection in some patients with intermediate-stage HCC. However, the prospective benefit of curative procedures applied after downstaging has not been tested to date. Furthermore, patients with intermediate-stage HCC represented a heterogeneous population because this classification included patients with wide ranges of tumor burden, liver function (Child-Pugh A or B), and disease etiology.^44^

The principal limitation of this study was its nonrandomized nature. However, the practical reasons that impaired our ability to perform randomized controlled trials included the differing levels of invasiveness of surgery versus TACE. In addition, most of the HCC patients possessed chronic HBV infection, which is not representative of all HCC patients worldwide. Therefore, larger, randomized, multicenter studies are needed to confirm out results.

In conclusion, surgical resection yielded better survival rates than TACE in intermediate (BCLC stage B) HCC patients with 1 to 3 tumor targets, but this approach produced similar results to TACE when >3 targets were present. Thus, because of the longer hospital stay and increased risk of more serious complications, surgical resection is only recommended for intermediate (BCLC stage B) HCC patients with 1 to 3 targets.
